# Retraction Note: Fe_3_O_4_@C@MCM41-guanidine core–shell nanostructures as a powerful and recyclable nanocatalyst with high performance for synthesis of Knoevenagel reaction

**DOI:** 10.1038/s41598-025-16433-3

**Published:** 2025-08-26

**Authors:** Aliyeh Barzkar, Alireza Salimi Beni

**Affiliations:** Department of Chemistry, Faculty of Science, Yasouj University, Yasouj, 75918-74831 Iran

Retraction of: *Scientific Reports* 10.1038/s41598-023-36352-5, published online 26 June 2023

The Editors have retracted this Article.

After publication, a number of concerns were raised. Specifically, two spectra in Figure 2 appear to overlap. Concerns were also raised regarding the physical validity and authenticity of the data presented in Figures 3 and 4.The Authors provided some source data, but this was insufficient to address the concerns raised. The Editors therefore no longer have confidence in the results and conclusions reported in this Article.

The Authors did not respond to correspondence from the Editors about this retraction.

